# Methamphetamine Causes Differential Alterations in Gene Expression and Patterns of Histone Acetylation/Hypoacetylation in the Rat Nucleus Accumbens

**DOI:** 10.1371/journal.pone.0034236

**Published:** 2012-03-28

**Authors:** Tracey A. Martin, Subramaniam Jayanthi, Michael T. McCoy, Christie Brannock, Bruce Ladenheim, Tiffany Garrett, Elin Lehrmann, Kevin G. Becker, Jean Lud Cadet

**Affiliations:** 1 Molecular Neuropsychiatry Research Branch, NIH/NIDA Intramural Research Program, Baltimore, Maryland, United States of America; 2 Gene Expression and Genomics Unit, Intramural Research Program, National Institute on Aging, National Institutes of Health, Baltimore, Maryland, United States of America; Institut National de la Santé et de la Recherche Médicale, France

## Abstract

Methamphetamine (METH) addiction is associated with several neuropsychiatric symptoms. Little is known about the effects of METH on gene expression and epigenetic modifications in the rat nucleus accumbens (NAC). Our study investigated the effects of a non-toxic METH injection (20 mg/kg) on gene expression, histone acetylation, and the expression of the histone acetyltransferase (HAT), ATF2, and of the histone deacetylases (HDACs), HDAC1 and HDAC2, in that structure. Microarray analyses done at 1, 8, 16 and 24 hrs after the METH injection identified METH-induced changes in the expression of genes previously implicated in the acute and longterm effects of psychostimulants, including immediate early genes and corticotropin-releasing factor (Crf). In contrast, the METH injection caused time-dependent decreases in the expression of other genes including Npas4 and cholecystokinin (Cck). Pathway analyses showed that genes with altered expression participated in behavioral performance, cell-to-cell signaling, and regulation of gene expression. PCR analyses confirmed the changes in the expression of c-fos, fosB, Crf, Cck, and Npas4 transcripts. To determine if the METH injection caused post-translational changes in histone markers, we used western blot analyses and identified METH-mediated decreases in histone H3 acetylated at lysine 9 (H3K9ac) and lysine 18 (H3K18ac) in nuclear sub-fractions. In contrast, the METH injection caused time-dependent increases in acetylated H4K5 and H4K8. The changes in histone acetylation were accompanied by decreased expression of HDAC1 but increased expression of HDAC2 protein levels. The histone acetyltransferase, ATF2, showed significant METH-induced increased in protein expression. These results suggest that METH-induced alterations in global gene expression seen in rat NAC might be related, in part, to METH-induced changes in histone acetylation secondary to changes in HAT and HDAC expression. The causal role that HATs and HDACs might play in METH-induced gene expression needs to be investigated further.

## Introduction

Addiction to methamphetamine (METH) is an international public health problem with an estimated 15–16 million users worldwide. The drug is abused because it is easy to manufacture and is cheaply available [1]. Acute administration of a range of METH doses results in a sense of euphoria, increased energy, and hypersexuality [2]. The acute effects can last for several hrs because of the long elimination half-life and the metabolite profiles of the drug [3]. Injection of the drug to rodents causes increased locomotor activity and stereotypic behaviors [4], [5] that are related, in part, to increased levels of dopamine (DA) in the synaptic cleft of brain regions such as the nucleus accumbens (NAC) and the striatum[6] that receive dopaminergic projections from midbrain DA neurons [7]. The drug also causes substantial changes in gene expression in some brain regions including the cortex, the dorsal striatum, and the midbrain [8], [9], [10], [11]. These molecular changes include transient increases and decreases in the expression of various transcription factors, neuropeptides, and genes that participate in several biological functions [8], [9], [10], [11], [12], [13].

Gene transcription is regulated by complex interactions of transcription factors with regulatory elements [14], [15]. During resting states, DNA is compacted in a way that interferes with the binding of transcription factors whereas DNA becomes more accessible during activation of cells by various stimuli [16]. DNA is indeed packaged into chromatin whose fundamental subunit, the nucleosome, is made of 4 core histones, histones H2A, H2B, H3, and H4 that form an octomer (2 of each histone) surrounded by 146 bp of DNA [17]. The N-tails of histones possess lysine residues that can be reversibly acetylated or deacetylated by several histone acetyltransferases (HATs) or by histone deacetylases (HDACs), respectively [18], [19]. Other histone modifications that can impact gene expression include methylation, phosphorylation and ubiquitylation [20], [21], [22]. These changes promote alterations in gene expression by modifying chromatin conformation and enabling or inhibiting recruitment of regulatory factors onto DNA sequences [23].

Therefore, the findings of METH-induced differential changes in gene expression had suggested to us that the drug might also cause changes in histone modifications. In the present study, we focused our attention on whether a single METH injection (20 mg/kg) that induces substantial changes in gene expression might also alter the status of histone acetylation in the rat nucleus accumbens (NAC). Herein, we report that METH administration does trigger time-dependent modifications in the acetylation of histones H3 and H4 as well as increases in ATF2 and HDAC2 expression in the NAC. We discuss the possibility that alterations in histone acetylation might, in part, influence METH-induced changes in gene expression in that brain structure. More direct evidence for a specific role of these histone modifications in mediating METH-induced effects will await further studies using chromatin immuprecipitation followed by massive parallel sequencing (ChIP-Seq) [24], [25].

## Results

### METH induces changes in gene expression in the NAC

**Figure 1:**
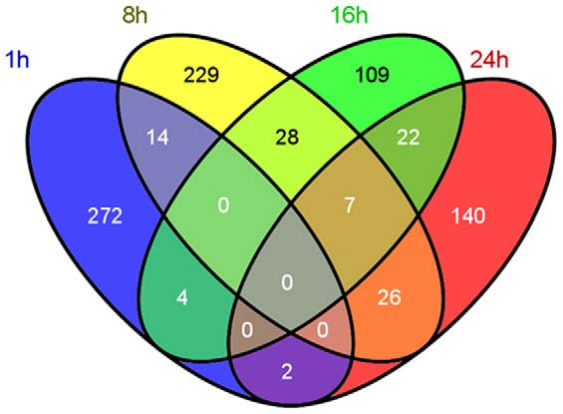
A single injection of METH (20 mg/kg) caused time-dependent changes in gene expression in the NAC. The Venn diagram depicts the overlap of genes identified in the four time points (1-hr, 8-hr, 16-hr and 24-hr) after administration of a single, non-toxic dose (20 mg/kg) of METH. RNA was extracted from NAC and the microarray experiments were performed as described in the methods section. Genes were identified as significantly changed if they show greater than ±1.7-fold changes at p<0.05.

**Figure 2:**
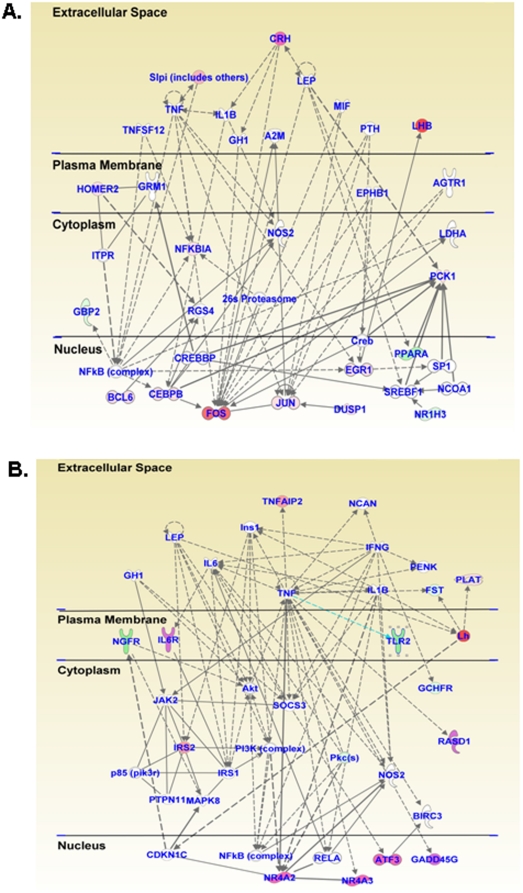
Networks of METH-induced changes in gene expression one hour after the drug injection. Networks of related genes were identified using Ingenuity Pathway Analysis (IPA) software. Figure 2A shows a network of affected genes that are involved in the control of gene expression, cellular growth and proliferation as well as endocrine functions while Fig. 2B shows genes that are involved in that are involved cell signaling, cellular development, and nervous system development. Relationships are shown as lines and arrows. Genes colored pink to red are up-regulated whereas those colored light to deep green are down-regulated, with the intensity of the color representing greater magnitude of changes.

We performed microarray analyses using Rat Illumina arrays that contain 22, 523 probes to identify genes that are differentially affected at 1, 8, 16, and 24 hrs after a single METH (20 mg/kg) injection that does not cause terminal degeneration in the rat [6]. As expected the METH injection caused significant changes in gene expression in the NAC. Figure 1 shows a Venn diagram depicting the overlap of genes that are altered by METH at the four time points. There were METH-induced changes in expression of 292 genes, with 165 being up-regulated and 127 being down-regulated at the 1-hr time point. IPA analyses revealed that they belong to classes of genes involved in behavioral responses, cell death, cellular development and morphology, cellular growth and proliferation, nervous system development and function, and regulation of gene expression. Figure 2A shows a network of affected genes that are involved in the control of gene expression, cellular growth and proliferation, as well as endocrine functions while Fig. 2B shows genes that are involved cell signaling, cellular development, and nervous system development. Genes of interest include corticotrophin releasing factor (Crf), early growth response 1 (Egr1), early growth response 2 (Egr2), c-fos, homer2, junB, neuronal PAS domain protein 4 (Npas4), nuclear receptor subfamily 4 (Nr4a3), among others (see Table S1 for a longer list).

**Figure 3:**
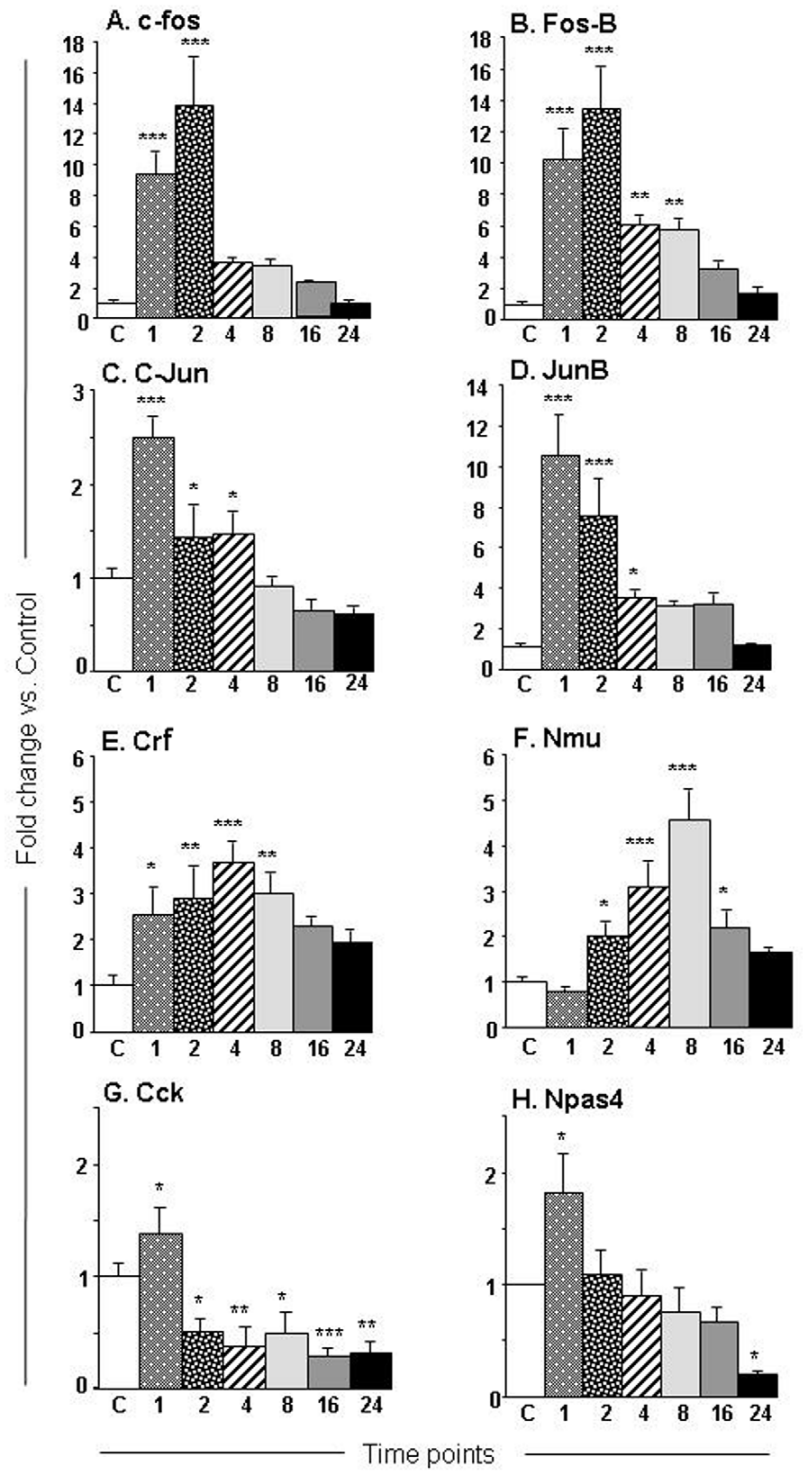
METH caused changes in the expression of transcription factors and neuropeptides in the NAC. The graphs show the effects of METH on transcript levels for (A) c-fos, (B) fosb, (C) c-jun, (D) junB, (E) Crf, (F) Nmu, (G) Cck, and (H) Npas4 mRNA levels at various time points after injection of the drug. The rats were injected with a single injection of METH as described in the method section. Total RNA was extracted from the NAC and used in qPCR assays. The relative amounts of transcripts were normalized to OAZ1 (ornithine decarboxylase antienzyme 1). Statistical significance was determined by ANOVA followed by post-hoc tests. Key to statistics (n = 5–8 animals per group): * = p<0.05; ** = p<0.01; *** = p<0.001, in comparison to the control group.

We used qPCR to confirm the changes in 4 members of the AP1 family of immediate early genes (IEGs). Figure 3 shows that the METH injection caused significant increases [F (6, 26) = 15.30, p<0.0001] in c-fos mRNA levels that were already apparent at 1-hr, peaked at 2-hr (13.6-fold) post-drug injection, and then tapered towards normal 24 hrs later (Fig. 3A). FosB expression also showed METH-mediated increases [F = 12.65, p<0.0001] which were obvious after 1-hr (10.2-fold), peaked at 2-hrs (13.4-fold) after the drug injection, and then reverted back to normal by 24-hr after the drug injection (Fig. 3B). METH caused smaller increases [F = 20.53; p<0.0001] in c-jun expression that peaked at 1-hr (2.5-fold) and then returned to normal levels 8 hrs later (Fig. 3C). There were also METH-induced increases [F = 15.55, p<0.0001] in junB mRNA levels which peaked at 1-hr (10.5-fold) and then tapered towards normal 24 hrs later (Fig. 3D).

In order to identify genes that were affected by METH at later time points, we also performed microarray analyses using tissues from animals euthanized at 8, 16, and 24 hrs after the METH injection. Figure 1 shows the overlap of differentially expressed genes at these three time points. Tables S2, S3, S4 show partial lists of the affected genes. In contrast to the 1-hr time point when more genes showed up-regulation by METH, there were more genes that were down-regulated from 8 to 24 hrs after the single METH injection. For example, there were a total of 304 differentially expressed genes at the 8-hr time point, with 151 being up-regulated and 153 being down-regulated by METH. Pathway analyses revealed that these genes participate in several molecular and cellular functions including cell-to-cell signaling, small molecule biochemistry, and cell death pathways. Some of the genes are also known to participate in behavioral performance, and tissue morphology. Figure S1 shows associated networks that include genes for lipid metabolism, molecular transport, and cellular compromise. Among the METH-regulated genes at the 8-hr time-point were Crf, follistatin (Fst), inhibin beta A (InhbA), and neuromedin U (Nmu) (Table S2). At the 16-hr time after drug injection, 170 genes were affected by METH, with 74 being up- and 96 being down-regulated. These genes are known to participate in several biological functions including protein synthesis, cell signaling, and nervous system development. Figure S2 shows a network that contains genes related to cellular assembly and organization, cellular movement, and nervous system function. At 16 hrs post-drug, genes of interest that show up-regulation included tumor necrosis factor superfamily alpha (TNF-alpha), and Nmu (Table S3). At the 24-hr time point, METH caused differential expression in 197 genes, with 63 being up- and 134 being down-regulated. These genes belong to classes of genes that participate in cell signaling, molecular transport, and nervous system development. Figure S3 shows a network that contain genes involved in cellular growth and proliferation, cell death, nervous system development, and behavior. Down-regulated genes include BDNF, Cck, Npas4, among others (Table S4). As shown in Figure 1, there were 14 genes that were affected at both the 1- and 8-hr time point. These genes that include Crf participate in endocrine system function, and lipid metabolism. There were 35 genes that were similarly affected at both 8- and 16-hr after the METH injection. IPA shows that these genes regulated cellular development, cellular compromise, and cell-to-cell signaling. In addition, 29 genes were similarly affected at the 16- and 24-hr time points. These are involved in cell-to-cell signaling, developmental disorders, molecular transport, and the regulation of gene expression.

Quantitative PCR confirmed the METH-induced changes in the expression of some genes of interest (Fig. 3). As shown in Fig. 3E, METH caused time-dependent increases [F (6, 30) = 3.39; p<0.0114] in Crf expression that was apparent at 1-hr (2.5-fold), peaked at 4-hr (3.7-fold), was still elevated at 8-hr (3-fold) and then began to revert towards normal thereafter. Figure 3F showed that METH caused significant changes [F = 14.36; p<0.0001] in the expression of NmU that appeared at 2-hr (2-fold), peaked at 8-hr (4.6-fold), and returned to normal values by 24-hr after the drug injection. Consistent with the microarray results, METH caused marked decreases [F (6, 29) = 8.72; p<0.001] in Cck expression in the nucleus accumbens (Fig. 3G). Interestingly, the decreases occurred as early as 2 hrs (−48%), remained low, and reached a nadir (−71%) by 16-hr after the drug injection. Figure 3H shows that the injection of METH caused significant changes [F (6, 27) = 4.65; p = 0.0023] in Npas4 expression. Post-hoc analyses revealed that METH caused rapid and very transient increases (1.8-fold) at 1-hr but delayed decreases (−81%) at the 24-hr time-point (Fig. 3H).

### METH-induces differential expression of acetylation status of histones H3 and H4 in the rat NAC

**Figure 4:**
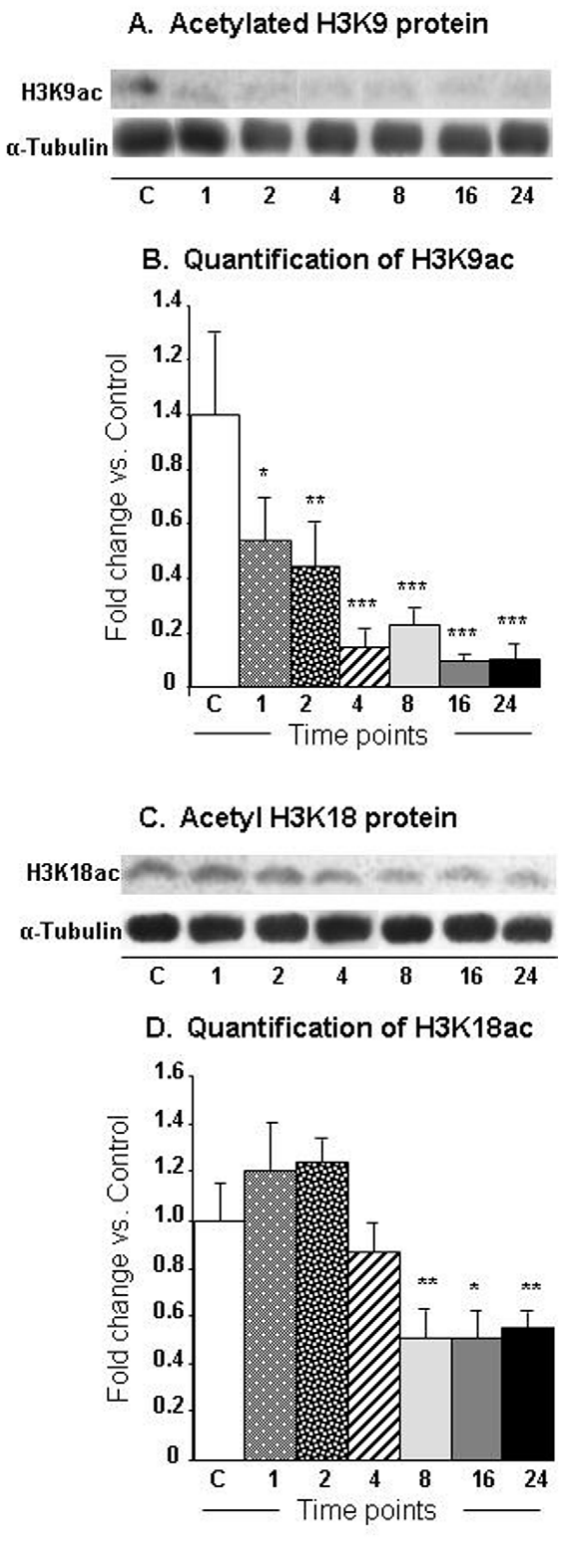
METH induced significant decreases in H3K9 and H3K18 acetylation in the NAC. The graphs show representative results from Western blot analyses using specific antibodies against (A) H3K9ac and H3K18ac (C) at various time points after injection of the drug. The rats were injected with a single injection of METH as described in the method section. Western blot analyses were carried out as described in the Method section. The relative amounts of proteins were normalized to tubulin. The bar graphs show quantification of the effects of METH on (B) H3K9ac and (D) H3K18ac, respectively. Statistical significance was determined by ANOVA followed by post-hoc tests. Key to statistics (n = 6 rats per group): * = p<0.05; ** = p<0.01; *** = p<0.001, in comparison to the control group.

**Figure 5:**
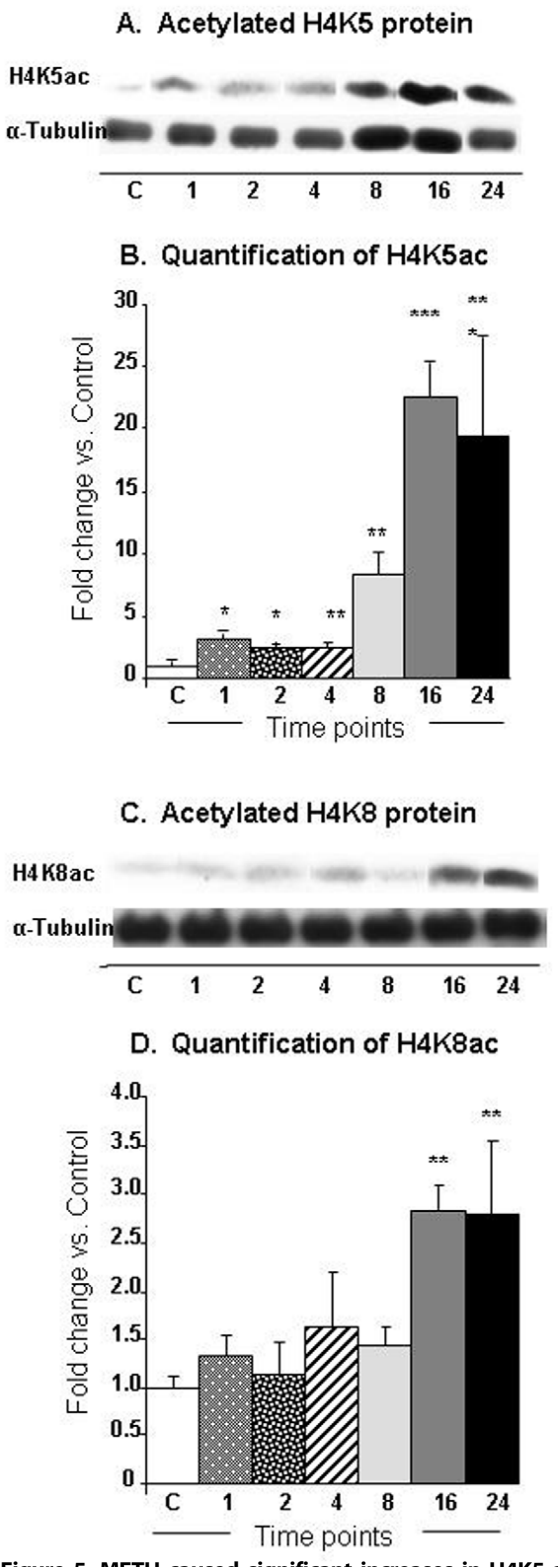
METH caused significant increases in H4K5 and H4K8 acetylation in the NAC. The graphs show representative results from Western blot analyses using specific antibodies against (A) H4K5ac and (C) H3K18 at various time points after injection of the drug. The rats were injected with a single injection of METH as described in the method section. Western blot analyses were carried out as described in the Method section. The relative amounts of proteins were normalized to tubulin. The bar graphs show quantification of the effects of METH on (B) H4K5ac and (D) H4K8ac, respectively. Statistical significance was determined by ANOVA followed by post-hoc tests. Key to statistics (n = 6 rats per group): * = p<0.05; ** = p<0.01; *** = p<0.001, in comparison to the control group.

Because changes in gene expression are regulated by histone modifications, in the present study, we tested the possibility that the METH injection might also cause rapid changes in histone acetylation that might parallel the changes in gene expression. We thus used western blot analyses to measure the acetylation status of histones H3 and H4 using specific antibodies against H3 acetylated at lysine 9 (H3K9ac) or at lysine 18 (H3K18ac) and against histone H4 acetylated at lysine 5 (H4K5ac), lysine 8 (H4K8ac), or lysine 16 (H4K16ac). The METH injection caused significant time-dependent decreases [F (6, 26) = 6.12, p<0.0004] in H3K9 acetylation in nuclear fractions of the NAC (Fig. 4A). The decreases were apparent at 1-hr (−54%), reached −85%, and persisted at the 24-hr time point (−90%) (Fig. 4B). The METH injections also caused significant decreases [F = 6.58, p<0.0001] in H3K18 acetylation (Fig. 4C). The decreases became obvious by 8-hr (−49%) and stayed at those low levels (Fig. 4D). Figure 5 shows that the METH injection was associated with significant increases [F (6, 30) = 18.24, p<0.0001] in H4K5 acetylation in these nuclear fractions (Fig. 5A). The increases occurred as early as 1-hr (279%), increased to 841% by 8 hrs, and were still very high (1904%) at 24 hrs after the METH injection (Fig. 5B). The METH injection also caused significant increases [F = 3.09, p = 0.0159] in H4K8 acetylation levels (Fig. 5C). The changes first appeared at 16 hrs (282%) and stayed elevated (279%) at the 24-hr time point (Fig. 5D). Figure S4 shows the effects of METH on the acetylation status of H4K16. The METH injection also caused significant decreases [F (6, 33) = 16.16, p<0.0001] in H4K16 acetylation levels (Fig. S4A). The changes first appeared at 1-hr (−97%) and stayed decreased (range of −85–96%) for 16 hrs, and reverted to normal at the 24-hr time point (Fig. S4B).

### METH-induced changes in HAT and HDAC protein levels in the NAC

**Figure 6:**
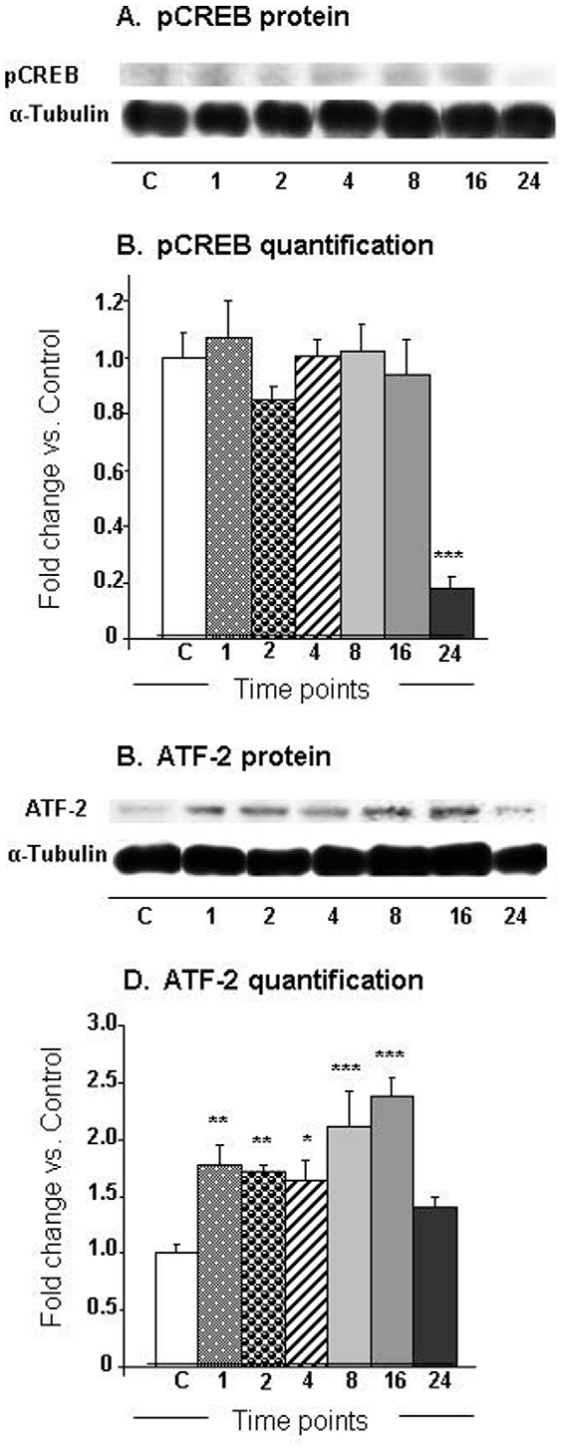
METH induced differential changes in the expression of (A) pCREB and (B) ATF2 in the NAC. The graphs show representative results from Western blot analyses using specific antibodies against (A) pCREB and (C) ATF2 at various time points after injection of the drug. Western blot analyses and statistical analyses were carried out as described above. The relative amounts of proteins were normalized to tubulin. The bar graphs show quantification of the effects of METH on (B) pCREB and (D) ATF2, respectively. Key to statistics (n = 6 rats per group): * = p<0.05; ** = p<0.01; *** = p<0.001, in comparison to the control group.

**Figure 7:**
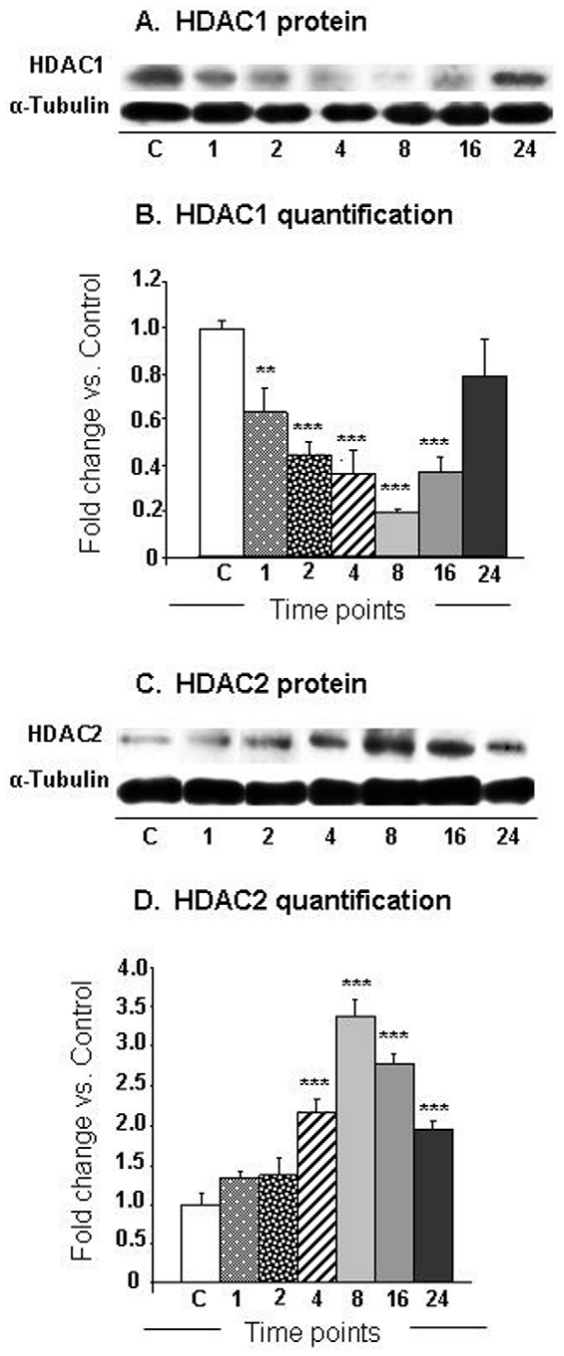
Differential METH-induced changes in the expression of (A) HDAC1 and (B) HDAC2 in the NAC. The graphs show representative results from Western blot analyses using specific antibodies against (A) HDAC1 and (C) HDAC2 at various time points after injection of the drug. Western blot analyses and statistical analyses were carried out as described above. The relative amounts of proteins were normalized to tubulin. The bar graphs show quantification of the effects of METH on (B) HDAC1 and (D) HDAC2, respectively. Key to statistics (n = 6 rats per group): * = p<0.05; ** = p<0.01; *** = p<0.001, in comparison to the control group.

Histone acetylation is regulated by a balance in HAT and HDAC expression [19], [26], [27]. In addition, gene induction by psychostimulants in the brain involves, in part, a cascade that includes phosphorylation of CREB to pCREB (see Cadet et al. [11] for review). Our analyses showed that METH caused significant changes [F (6, 17) = 9.48, p<0.0001] in pCREB protein expression, with prominent decreases occurring at the 24-hr time point (−83%) (Fig. 6A and 6B). Because ATF2, a member of the ATF/CREB family, has been reported to cause acetylation of histone H4 [28], we also measured the protein levels of ATF2. Figure 6C shows that METH caused significant increases [F (6, 16) = 7.49, p = 0.0006] in ATF2 protein levels. The increases were apparent as early as 1-hr (179%), peaked at 16 hrs (239%), and then normalized by 24 hrs after the single METH injection (Fig. 6D). Because METH caused decreased acetylation of histone H3K9, H3K18, and H4K16, we also measured the protein expression of HDAC1 and HDAC2 in nuclear fractions of the NAC (Fig. 7). Figure 7A shows the effects of METH on HDAC1 expression. There were significant METH-induced decreases [F (6, 18) = 7.76, p = 0.0003] in HDAC1 protein levels that were apparent at 1-hr (−39%), reached the lowest levels (−81%) at 8 hrs, and returned towards normal (−20%) at 24 hrs after the injection (Fig. 7B). Figure 7C shows that there were significant increases [F (6, 17) = 31.03; p<0.0001] in HDAC2 protein levels that were first apparent at 4 hrs (217%), reached a peak at 8 hrs (339%) and were still elevated (197%) at 24 hrs after the drug injection (Fig. 7D).

## Discussion

Recent studies have shown that a single administration of METH can have prolonged behavioral, biochemical, and molecular effects in rodents [6], [12], [13], [29]. However, the molecular underpinnings for these long-term effects of this drug have yet to be fully elucidated. As an initial step in a program of studies to clarify the molecular bases of METH-induced complex changes in the brain, the present study investigated the time course of potential METH-induced changes in gene expression and histone modifications in the NAC, a structure that is closely tied to the behavioral effects of psychostimulants [30]. We thus used a dose of METH that does not cause any persistent toxicity in the brain [6] to measure potential prolonged global changes in gene expression in that structure.

Among other genes, we identified Crf as one gene that was substantially induced in the NAC. CRF, a polypeptide composed of 41 amino acids, is the major regulator of the pituitary-adrenal axis [31]. CRF has been shown to trigger various biochemical and behavioral changes in animals [32], [33], [34]. For example, infusion of CRF by itself into the NAC caused increased locomotor activity [35]. The behavioral effects are most probably due to the widespread distribution of CRF and of its receptors in the mammalian brain [36], [37], [38], [39]. The acute METH-induced increases in Crf expression are interesting because dysregulation of processes regulated by CRF have been implicated in addiction to illicit drugs [32], [40]. Our results are also consistent with the report that binge cocaine administration can also cause increases in Crf in rat brain [41] and suggest that psychostimulants of diverse classes can cause increases in Crf mRNA levels. Of related interest, low doses of CRF can potentiate stereotypic behaviors caused by amphetamine [42], suggesting an additional role of CRF in behaviors induced by amphetamine analogs. Cocaine was also shown to induce CRF receptor-dependent hyperlocomotion [43]. Importantly, Moffett and Goeders (2007) [44] recently reported that CP-154–526, a CRF type-1 receptor antagonist, was able to attenuate METH-induced reinstatement of extinguished METH-seeking behaviors in the rat. The METH-induced increases in CRF expression might be related to the fact that the drug injection caused downregulation of HDAC1 expression in the nuclear fraction of the NAC since HDAC1 up-regulation has been reported to cause downregulation of CRF expression [45].

In contrast to the METH-increases in Crf mRNA levels, we found substantial decreases in Cck mRNA levels that lasted through the duration of the experiments. The neuropeptide, CCK, is synthesized as a pre-pro-hormone that is cleaved to produce several peptides [46] including the main neurotransmitter, CCK-8, which is involved in a number of behavioral and biological functions [47]. CCK is widely distributed in the rodent brain [48], [49] and co-localizes with several neurotransmitters including dopamine [50], [51]. CCK is located within vesicles [49] from which it can be released [52]. There also exists an interesting literature on the role of CCK on dopamine-mediated behaviors, with different subregions of the NAC showing differential effects on DA-mediated locomotor activity [53]. For example, Crawley et al. (1985) [54] reported that injection of DA into the NAC caused dose-dependent increases in locomotor activity. Injection of CCK alone had no effects on locomotor responses. However, co-administration of CCK with DA potentiated DA-induced hyperlocomotion. Subsequent studies revealed involvement of CCK-A and CCK-B receptors in the mediation of DA-induced hyperlocomotion in the medial posterior NAC and anterior NAC, respectively [55]. On the other hand, Weiss et al., (1988) [56] reported that injections of CCK into the NAC antagonized amphetamine-induced hyperlocomotion. CCK also antagonized apomorphine-induced hyperlocomotion [57]. Nevertheless, our observations of METH-induced decreases in Cck mRNA levels suggest that injection of METH might have caused co-release of CCK and DA from midbrain projections to the NAC [50] and that local CCK-containing cell bodies [48], [49] might have compensated to the overstimulation of CCK receptors by decreasing Cck transcript levels. This idea is consistent with the report that amphetamine caused DA release that was accompanied with a rapid and transient CCK release in the NAC with a peak response within the first 20 min after the drug injection [58]. The timing of the AMPH-induced release of CCK is consistent with the decreases in Cck mRNA levels and with the suggested possibility that the observed decreased Cck transcript levels are potentially compensatory to METH-induced CCK release. Our results are also consistent with the report that a single dose of a lower dose of METH (0.5 mg/kg) [59] or daily METH injections for 14 days [60] caused decreases in Cck mRNA levels in the rat brain.

The accumulated evidence indicates that increases in gene expression are associated with increases in histone acetylation whereas decreases in gene expression correlate with hypoacetylation of histones [61], [62]. Histone H4 acetylation enables regulatory proteins to access DNA and plays a major role in regulating gene expression [63], [64]. The substantial METH-induced increases of histone H4K5 and H4K8 acetylation are, thus, of interest because both H4K5 and H4K8 belong to the class of so-called “common modification module” that is present on active and poised promoters [65], thus suggesting that METH might have influenced the composition of this module. The increases in H4K5 and H4K8 acetylation might be secondary to the METH-induced increases in the expression of ATF2 that has HAT activity [66] for histone 4 [28]. ATF-2 is a member of the ATF/CREB family of transcription factors that contain a common basic region leucine zipper (bZIP) [67]. The family members that include ATF1, ATF2, ATF3, ATF4, and CREB, among others can homodimerize or heterodimerize with members of other bZIP-containing proteins including members of the AP1 family of transcription factor [68]. ATF-2 itself, can heterodimerize with c-Jun [69] and c-Fos [70]. ATF-2 has been shown to bind to both the AP-1 site (TGACTCA) and the CRE site (TG/AACGTCA) [68], [71]. As mentioned above, ATF-2 possesses intrinsic histone acetyltransferase (HAT) activity on histone H4 and promotes CRE-dependent transcription [72]. A potential role for ATF2 in METH-induced H4 acetylation is consistent with our findings that the increases in ATF2 preceded H4K5 and H4K8 acetylation after the METH injection. Because ATF2 HAT activity has been shown to be necessary for CRE-dependent transcription [72], it is fair to propose that the delayed increases in gene expression after a single injection of METH might be due, in part, to increases in H4K5 and H4K8 acetylation secondary to METH-induced increases in ATF2 expression. It is important to note that the METH-induced increased in H4K5 acetylation could also be due to the drug-induced decreases in HDAC1 expression observed in the nuclear fractions obtained from the NAC because RNAi-mediated reduction in HDAC1 nuclear staining was reported to induce increases in H4K5 acetylation [73]. The authors also reported that RNAi-induced decreases in HDAC1 expression were accompanied by increased HDAC2 expression, which we also observed after the single injection of METH (Fig. 7C). Although the specific role for ATF2, HDAC1, and H4K5ac in the regulation of specific target genes will have to depend on future studies using chromatin precipitation followed by massive parallel sequencing (ChIP-Seq) in order to provide genome-scale quantification of ATF2- and HDAC1-, and H4K5ac-DNA binding [24], [25], these investigations are beyond the scope of the present paper. Nevertheless, it is notable that some known ATF2 target genes including c-fos and c-jun [74], [75] did show METH-induced increases (see Figure 3B and 3C, respectively). Similarly, CRF whose expression is downregulated by increased HDAC1 expression [45] showed significant increases (Fig. 3E) after the METH injection that caused decreases in HDAC1 expression in the nuclear fraction of the NAC (see Fig. 7A). These caveats, notwithstanding, our present observations provide additional support for the proposal that changes in gene regulation that occur after exposure to illicit substance such as cocaine might be consequences of alterations in histone modifications [76], [77]. Future studies in this laboratory will seek to determine the role of specific histone modifications in acute and chronic METH-induced changes in gene expression in the brain.

Our experiments also showed that METH caused substantial time-dependent decreases in the acetylation of histone H3K9, H3K18, and H4K16. These changes in histone acetylation are consistent with our demonstration that METH also caused substantial increases in HDAC2 protein levels in the NAC. Indeed, recruitment of HDAC proteins to the promoters of genes is known to cause histone hypoacetylation and subsequent transcriptional repression of various genes [78], [79], including neuronal genes [80], [81]. Therefore, the observed METH-induced histone hypoacetylation in H3K9 and H3K18 might offer a partial explanation for the METH-induced repression of many genes observed at 8, 16, and 24 hrs after the single METH injection, with a larger number of genes being down-regulated than up-regulated at the 8-hr, 16-hr and 24-hr time points. Although previous studies have focused mainly on the role of the cAMP/PKA/CREB pathway [82] in psychostimulant-induced changes in gene expression [11], the present study identifies increases in HDAC2, but decreased H3K9ac and H3K18ac expression as potentially effectors of METH-induced repression of genes that were affected both early and late during the course of the present experiments. This idea is consistent with the report that HDAC inhibition caused potentiation of kainate-induced increases in c-fos and c-jun expression [83]. Finally, the greater number of repressed genes observed at the 24-hr time point might be due, in part, to the concomitant METH-induced decreases in pCREB expression that occurred at that time.

In summary, our data identify, for the first time, the existence of concomitant increases and decreases in the expression of many genes in the rat NAC after a single METH injection. These changes in gene expression were associated with diverse alterations in histone acetylation. These observations are consistent with previous demonstrations that histone modifications can participate in cross-talks [84], [85] that regulate gene expression in a complex fashion [65], [86], [87]. Our findings also suggest that studies of the effects of psychostimulants need to take into consideration the combinatorial roles of histones in their control of gene expression [65]. Our study also supports the notion that modulators of histone acetylation might be attractive therapeutic players against various diseased states including addiction to licit and illicit substances. Although more studies are needed to determine the specific manner by which METH-induced changes in ATF2, HDACs, and histone acetylation might differentially influence the levels of specific transcripts, our data suggest that a single METH exposure can cause profound alterations in the molecular machinery of the brain. Finally, even though the present study aimed to provide an initial look at the effects of METH on histone acetylation, future studies will investigate the effects of the drug on other histone modifications because understanding the molecular impact of these combinatorial alterations might lead to better therapeutic strategies against the adverse effects of psychostimulant use.

## Materials and Methods

### Animals and Drug treatment

Male Sprague-Dawley rats (Charles River Labs, Raleigh, NC, USA), weighing 375±25 g, were used in the experiments. Rats were housed in a temperature-controlled (22.2+0.2°C) room with free access to food and water. The animals received a single injection of METH (20 mg/kg) and were euthanized at various time points afterwards. This METH dose has been used in behavioral and biochemical experiments and has been shown robust increases in DA release in the NAC [6] (6). All animal use procedures were according to the NIH Guide for the Care and Use of Laboratory Animals and were approved by the National Institute of Drug Abuse-/Intramural Research Program (IRP) Animal Care and Use Committee (NIDA/IRP-ACUC).

### Tissue collection and RNA extraction

At the indicated time after the METH or saline injections, rats (n = 5 per group) were euthanized and the NAC was dissected and immediately put on dry ice. Total RNA was isolated from the nucleus accumbens according to the manufacturer's manual using Qiagen RNeasy mini kit (Qiagen, Valencia, CA, USA). RNA integrity was detected using an Agilent 2100 Bioanalyzer (Agilent, Palo Alto, CA, USA).

### Microarray hybridization and data analysis

Microarray hybridization was carried out using RatRef-12 Expression BeadChips arrays (22, 523 probes) (Illumina Inc., San Diego, CA) essentially as previously described by us [13], [88]. Raw data were imported into GeneSpring and normalized using global normalization. The normalized data were used to identify changes in gene expression at the different time points (1, 2, 4, 8, 16 and 24 hr) after the injection of METH. A gene was identified as significantly affected if it showed increased or decreased expression according to an arbitrary cut-off of 1.7-fold changes at p<0.05, according to the GeneSpring statistical package. Similar criteria have been successfully used in our previous microarray studies [13], [88], [89]. Network analyses were performed using the Ingenuity Pathway Analysis (IPA) software (Ingenuity Systems, Redwood City, CA). The IPA software allows for the identification of networks, canonical pathways, and biological functions that are affected by the drug. We also used the IPA software to graphically show the cellular location of genes significantly affected by METH and to overlay differentially affected networks of interest.

### Quantitative polymerase chain reaction (qPCR)

Total RNA was reverse-transcribed to cDNA with oligo dT primer using Advantage RT for PCR kits (BD Biosciences Clontech Laboratories, Palo Alto, CA, USA). Gene-specific primers were generated using Light Cycler Probe Design software and synthesized by the Synthesis and Sequencing Facility of Johns Hopkins University (Baltimore, MD, USA). Real-time PCR experiments were performed using iQ SYBR Green supermix (Bio-Rad Laboratories, Hercules, CA) and Chromo 4 Detector (MJ Research, Inc. Waltham, MA. For all qPCR experiments, individual data (n = 5–8 per group) were normalized using the corresponding OAZ1 signal as described [90].

### Western Blot Analysis

Western blot analyses were carried out essentially as previously described by our laboratory [13]. The antibodies used were anti-acetyl-histone H4K8 (Lys 8), H4K12 (Lys 12), and H4K16 (Lys 16) (Millipore, Billirica, MA, USA); anti-HDAC1 and anti-HDAC2 (Santa Cruz Biotechnology, Inc., Santa Cruz, CA, USA); anti-phospho-CREB and anti-ATF2 (Cell Signaling Technology, Danvers, MA, USA). To confirm equal protein loading, blots were re-probed with α-tubulin antibody (1∶4000; Sigma, 2 h at RT). LumiGLO chemiluminescent reagents (Cell Signaling Technology Inc., Danvers, MA, USA) were used to detect protein expression. Signal intensity was measured densitometrically with LabWorks version 4.5 (BioImaging Systems analysis software, BioImaging System, UVP Inc., Upland, CA). For quantification, the signal intensity was normalized using the signal intensity of tubulin (n = 6 per group).

### Statistical Analyses

Data for quantitative PCR and Western blot analyses are presented as means ± SEM. Statistical analyses were performed using one-way ANOVA analysis followed by Fisher's protected least significant difference (StatView 4.02, SAS Institute, Cary, NC). Criteria for significance were set at p≤0.05.

## Supporting Information

Figure S1 **A network of genes whose expression was affected by METH at 8-hr after the injection of the drug.** Networks of related genes were identified using Ingenuity Pathway Analysis (IPA) software. The figure shows a network of affected genes that are involved in lipid metabolism, molecular transport, and cellular compromise and cell death. Color schemes are as described in figure 2.
(PPT)

Figure S2 **A network of genes whose expression was affected by METH at 16-hr after the injection of the drug.** Networks of related genes were identified using Ingenuity Pathway Analysis (IPA) software. The figure shows a network of genes that participate in cellular development, cellular growth and nervous system development. Relationships are shown as lines and arrows. Color schemes are as described in figure 2.
(PPT)

Figure S3 **A network of genes whose expression was affected by METH at 24-hr after injection of the drug.** Networks of related genes were identified using Ingenuity Pathway Analysis (IPA) software. The figure shows genes involved in the regulation of cell death, nervous system development, and cell proliferation. Relationships are shown as lines and arrows. Color schemes are as described in figure 2.
(PPT)

Figure S4 **METH administration caused significant decreases in H4K16 acetylation in the NAC.** The graph shows representative results from Western blot analyses using a specific antibody against (A) H4K16ac at various time points after the injection of the drug. Western blot analyses and statistical analyses were carried out as described in Fig. 4. The bar graph shows quantification of the effects of METH on H4K16ac. Key to statistics: * = p<0.05; ** = p<0.01; *** = p<0.001, in comparison to the control group.
(PPT)

Table S1 **Partial list of METH-upregulated genes measured at 1-hr after the drug injection.** The list of genes was generated as described in the text. The genes are listed in descending order according to METH-induced fold changes in transcript levels.
(DOC)

Table S2 **Partial list of METH-regulated genes measured at 8-hr after the drug injection.** The genes are listed in descending order according to METH-induced fold changes in gene expression at the 8-hr. time point. The values for the 16- and 24-hr time points are listed for comparison.
(DOC)

Table S3 **Partial list of METH-regulated genes measured at 16-hr after the drug injection.** The genes are listed in descending order according to METH-induced fold changes in gene expression at the 16-hr. time point. The values for the 8- and 24-hr time points are listed for comparison.
(DOC)

Table S4 **Partial list of METH- regulated genes measured at 24-hr after the drug injection.** The genes are listed in descending order according to METH-induced fold changes in gene expression at the 24-hr. time point. The values for the 8- and 16-hr time points are listed for comparison.
(DOC)

## References

[1] Krasnova IN, Cadet JL (2009). Methamphetamine toxicity and messengers of death.. Brain Res Rev.

[2] Barr AM, Panenka WJ, MacEwan GW, Thornton AE, Lang DJ (2006). The need for speed: an update on methamphetamine addiction.. J Psychiatry Neurosci.

[3] Schepers RJ, Oyler JM, Joseph RE, Cone EJ, Moolchan ET (2003). Methamphetamine and amphetamine pharmacokinetics in oral fluid and plasma after controlled oral methamphetamine administration to human volunteers.. Clin Chem.

[4] Frankel PS, Hoonakker AJ, Danaceau JP, Hanson GR (2007). Mechanism of an exaggerated locomotor response to a low-dose challenge of methamphetamine.. Pharmacol Biochem Behav.

[5] Hall DA, Stanis JJ, Marquez Avila H, Gulley JM (2008). A comparison of amphetamine- and methamphetamine-induced locomotor activity in rats: evidence for qualitative differences in behavior.. Psychopharmacology (Berl).

[6] Xi ZX, Kleitz HK, Deng X, Ladenheim B, Peng XQ (2009). A single high dose of methamphetamine increases cocaine self-administration by depletion of striatal dopamine in rats.. Neuroscience.

[7] Bjorklund A, Dunnett SB (2007). Dopamine neuron systems in the brain: an update.. Trends Neurosci.

[8] Cadet JL, Krasnova IN, Ladenheim B, Cai NS, McCoy MT (2009). Methamphetamine preconditioning: differential protective effects on monoaminergic systems in the rat brain.. Neurotox Res.

[9] Cadet JL, Jayanthi S, McCoy MT, Vawter M, Ladenheim B (2001). Temporal profiling of methamphetamine-induced changes in gene expression in the mouse brain: Evidence from cDNA array.. Synapse.

[10] Thomas DM, Francescutti-Verbeem DM, Liu X, Kuhn DM (2004). Identification of differentially regulated transcripts in mouse striatum following methamphetamine treatment–an oligonucleotide microarray approach.. J Neurochem.

[11] Cadet JL, Jayanthi S, McCoy MT, Beauvais G, Cai NS (2010). Dopamine D1 Receptors, Regulation of Gene Expression in the Brain, and Neurodegeneration.. CNS Neurol Disord Drug Targets.

[12] Jayanthi S, Deng X, Ladenheim B, McCoy MT, Cluster A (2005). Calcineurin/NFAT-induced up-regulation of the Fas ligand/Fas death pathway is involved in methamphetamine-induced neuronal apoptosis.. Proc Natl Acad Sci U S A.

[13] Jayanthi S, McCoy MT, Beauvais G, Ladenheim B, Gilmore K (2009). Methamphetamine induces dopamine D1 receptor-dependent endoplasmic reticulum stress-related molecular events in the rat striatum.. PLoS One.

[14] Belotserkovskaya R, Saunders A, Lis JT, Reinberg D (2004). Transcription through chromatin: understanding a complex FACT.. Biochim Biophys Acta.

[15] Razin SV, Iarovaia OV, Sjakste N, Sjakste T, Bagdoniene L (2007). Chromatin domains and regulation of transcription.. J Mol Biol.

[16] Beato M (1996). Chromatin structure and the regulation of gene expression: remodeling at the MMTV promoter.. J Mol Med (Berl).

[17] Rando OJ, Ahmad K (2007). Rules and regulation in the primary structure of chromatin.. Curr Opin Cell Biol.

[18] Campos EI, Reinberg D (2009). Histones: annotating chromatin.. Annu Rev Genet.

[19] Morrison BE, Majdzadeh N, D'Mello SR (2007). Histone deacetylases: focus on the nervous system.. Cell Mol Life Sci.

[20] Berger SL (2010). Cell signaling and transcriptional regulation via histone phosphorylation.. Cold Spring Harb Symp Quant Biol.

[21] Hublitz P, Albert M, Peters AH (2009). Mechanisms of transcriptional repression by histone lysine methylation.. Int J Dev Biol.

[22] Osley MA, Fleming AB, Kao CF (2006). Histone ubiquitylation and the regulation of transcription.. Results Probl Cell Differ.

[23] Verdone L, Agricola E, Caserta M, Di Mauro E (2006). Histone acetylation in gene regulation.. Brief Funct Genomic Proteomic.

[24] MacQuarrie KL, Fong AP, Morse RH, Tapscott SJ (2011). Genome-wide transcription factor binding: beyond direct target regulation.. Trends Genet.

[25] Northrup DL, Zhao K (2011). Application of ChIP-Seq and related techniques to the study of immune function.. Immunity.

[26] Keppler BR, Archer TK (2008). Chromatin-modifying enzymes as therapeutic targets–Part 2.. Expert Opin Ther Targets.

[27] Megee PC, Morgan BA, Mittman BA, Smith MM (1990). Genetic analysis of histone H4: essential role of lysines subject to reversible acetylation.. Science.

[28] Bruhat A, Cherasse Y, Maurin AC, Breitwieser W, Parry L (2007). ATF2 is required for amino acid-regulated transcription by orchestrating specific histone acetylation.. Nucleic Acids Res.

[29] Thiriet N, Deng X, Solinas M, Ladenheim B, Curtis W (2005). Neuropeptide Y protects against methamphetamine-induced neuronal apoptosis in the mouse striatum.. J Neurosci.

[30] Luscher C, Malenka RC (2011). Drug-evoked synaptic plasticity in addiction: from molecular changes to circuit remodeling.. Neuron.

[31] Vale W, Spiess J, Rivier C, Rivier J (1981). Characterization of a 41-residue ovine hypothalamic peptide that stimulates secretion of corticotropin and beta-endorphin.. Science.

[32] Koob GF, Zorrilla EP (2010). Neurobiological mechanisms of addiction: focus on corticotropin-releasing factor.. Curr Opin Investig Drugs.

[33] Morley JE, Levine AS (1982). Corticotrophin releasing factor, grooming and ingestive behavior.. Life Sci.

[34] Shalev U, Erb S, Shaham Y (2010). Role of CRF and other neuropeptides in stress-induced reinstatement of drug seeking.. Brain Res.

[35] Holahan MR, Kalin NH, Kelley AE (1997). Microinfusion of corticotropin-releasing factor into the nucleus accumbens shell results in increased behavioral arousal and oral motor activity.. Psychopharmacology (Berl).

[36] De Souza EB, Insel TR, Perrin MH, Rivier J, Vale WW (1985). Corticotropin-releasing factor receptors are widely distributed within the rat central nervous system: an autoradiographic study.. J Neurosci.

[37] Perrin MH, Vale WW (1999). Corticotropin releasing factor receptors and their ligand family.. Ann N Y Acad Sci.

[38] Primus RJ, Yevich E, Baltazar C, Gallager DW (1997). Autoradiographic localization of CRF1 and CRF2 binding sites in adult rat brain.. Neuropsychopharmacology.

[39] Sawchenko PE, Imaki T, Potter E, Kovacs K, Imaki J (1993). The functional neuroanatomy of corticotropin-releasing factor.. Ciba Found Symp.

[40] Sarnyai Z, Shaham Y, Heinrichs SC (2001). The role of corticotropin-releasing factor in drug addiction.. Pharmacol Rev.

[41] Zhou Y, Spangler R, LaForge KS, Maggos CE, Ho A (1996). Corticotropin-releasing factor and type 1 corticotropin-releasing factor receptor messenger RNAs in rat brain and pituitary during “binge”-pattern cocaine administration and chronic withdrawal.. J Pharmacol Exp Ther.

[42] Cole BJ, Koob GF (1989). Low doses of corticotropin-releasing factor potentiate amphetamine-induced stereotyped behavior.. Psychopharmacology (Berl).

[43] Lu L, Liu Z, Huang M, Zhang Z (2003). Dopamine-dependent responses to cocaine depend on corticotropin-releasing factor receptor subtypes.. J Neurochem.

[44] Moffett MC, Goeders NE (2007). CP-154,526, a CRF type-1 receptor antagonist, attenuates the cue-and methamphetamine-induced reinstatement of extinguished methamphetamine-seeking behavior in rats.. Psychopharmacology (Berl).

[45] Miller L, Foradori CD, Lalmansingh AS, Sharma D, Handa RJ (2011). Histone deacetylase 1 (HDAC1) participates in the down-regulation of corticotropin releasing hormone gene (crh) expression.. Physiol Behav.

[46] Beinfeld MC (2003). Biosynthesis and processing of pro CCK: recent progress and future challenges.. Life Sci.

[47] Crawley JN, Corwin RL (1994). Biological actions of cholecystokinin.. Peptides.

[48] Zaborszky L, Alheid GF, Beinfeld MC, Eiden LE, Heimer L (1985). Cholecystokinin innervation of the ventral striatum: a morphological and radioimmunological study.. Neuroscience.

[49] Vanderhaeghen JJ, Lotstra F, Demey J, Gilles C (1980). Immunohistochemical Localization of Cholecystokinin-Like and Gastrin-Like Peptides in the Brain and Hypophysis of the Rat.. Proceedings of the National Academy of Sciences of the United States of America-Biological Sciences.

[50] Seroogy KB, Dangaran K, Lim S, Haycock JW, Fallon JH (1989). Ventral mesencephalic neurons containing both cholecystokinin- and tyrosine hydroxylase-like immunoreactivities project to forebrain regions.. J Comp Neurol.

[51] Seroogy KB, Mehta A, Fallon JH (1987). Neurotensin and cholecystokinin coexistence within neurons of the ventral mesencephalon: projections to forebrain.. Exp Brain Res.

[52] Emson PC, Lee CM, Rehfeld JF (1980). Cholecystokinin octapeptide: vesicular localization and calcium dependent release from rat brain in vitro.. Life Sci.

[53] Vaccarino FJ, Rankin J (1989). Nucleus accumbens cholecystokinin (CCK) can either attenuate or potentiate amphetamine-induced locomotor activity: evidence for rostral-caudal differences in accumbens CCK function.. Behav Neurosci.

[54] Crawley JN (1985). Cholecystokinin potentiates dopamine-mediated behaviors in the nucleus accumbens, a site of CCK-DA co-existence.. Psychopharmacol Bull.

[55] Crawley JN (1992). Subtype-selective cholecystokinin receptor antagonists block cholecystokinin modulation of dopamine-mediated behaviors in the rat mesolimbic pathway.. J Neurosci.

[56] Weiss F, Tanzer DJ, Ettenberg A (1988). Opposite actions of CCK-8 on amphetamine-induced hyperlocomotion and stereotypy following intracerebroventricular and intra-accumbens injections in rats.. Pharmacol Biochem Behav.

[57] Weiss F, Ettenberg A, Koob GF (1989). CCK-8 injected into the nucleus accumbens attenuates the supersensitive locomotor response to apomorphine in 6-OHDA and chronic-neuroleptic treated rats.. Psychopharmacology (Berl).

[58] Hurd YL, Lindefors N, Brodin E, Brene S, Persson H (1992). Amphetamine regulation of mesolimbic dopamine/cholecystokinin neurotransmission.. Brain Res.

[59] Yoshikawa T, Kunishima C, Yamada K, Nabeshima T, Shibuya H (1994). Effect of a single injection of psychoactive drugs on CCK mRNA in rat brain.. Peptides.

[60] Fukamauchi F (1996). Changes in cholecystokinin mRNA expression in methamphetamine-induced behavioral sensitization.. Neurochem Int.

[61] Mutskov V, Gerber D, Angelov D, Ausio J, Workman J (1998). Persistent interactions of core histone tails with nucleosomal DNA following acetylation and transcription factor binding.. Mol Cell Biol.

[62] Ura K, Kurumizaka H, Dimitrov S, Almouzni G, Wolffe AP (1997). Histone acetylation: influence on transcription, nucleosome mobility and positioning, and linker histone-dependent transcriptional repression.. EMBO J.

[63] Lee DY, Hayes JJ, Pruss D, Wolffe AP (1993). A positive role for histone acetylation in transcription factor access to nucleosomal DNA.. Cell.

[64] Rundlett SE, Carmen AA, Suka N, Turner BM, Grunstein M (1998). Transcriptional repression by UME6 involves deacetylation of lysine 5 of histone H4 by RPD3.. Nature.

[65] Wang Z, Zang C, Rosenfeld JA, Schones DE, Barski A (2008). Combinatorial patterns of histone acetylations and methylations in the human genome.. Nat Genet.

[66] Kawasaki H, Schiltz L, Chiu R, Itakura K, Taira K (2000). ATF-2 has intrinsic histone acetyltransferase activity which is modulated by phosphorylation.. Nature.

[67] Hai T, Hartman MG (2001). The molecular biology and nomenclature of the activating transcription factor/cAMP responsive element binding family of transcription factors: activating transcription factor proteins and homeostasis.. Gene.

[68] van Dam H, Castellazzi M (2001). Distinct roles of Jun : Fos and Jun : ATF dimers in oncogenesis.. Oncogene.

[69] De Cesare D, Vallone D, Caracciolo A, Sassone-Corsi P, Nerlov C (1995). Heterodimerization of c-Jun with ATF-2 and c-Fos is required for positive and negative regulation of the human urokinase enhancer.. Oncogene.

[70] Hai T, Curran T (1991). Cross-family dimerization of transcription factors Fos/Jun and ATF/CREB alters DNA binding specificity.. Proc Natl Acad Sci U S A.

[71] Abdel-Hafiz HA, Heasley LE, Kyriakis JM, Avruch J, Kroll DJ (1992). Activating transcription factor-2 DNA-binding activity is stimulated by phosphorylation catalyzed by p42 and p54 microtubule-associated protein kinases.. Mol Endocrinol.

[72] Kawasaki H, Taira K, Yokoyama K (2000). Histone acetyltransferase (HAT) activity of ATF-2 is necessary for the CRE-dependent transcription.. Nucleic Acids Symp Ser.

[73] Ma P, Schultz RM (2008). Histone deacetylase 1 (HDAC1) regulates histone acetylation, development, and gene expression in preimplantation mouse embryos.. Dev Biol.

[74] Liu H, Deng X, Shyu YJ, Li JJ, Taparowsky EJ (2006). Mutual regulation of c-Jun and ATF2 by transcriptional activation and subcellular localization.. EMBO J.

[75] Lopez-Bergami P, Lau E, Ronai Z (2010). Emerging roles of ATF2 and the dynamic AP1 network in cancer.. Nat Rev Cancer.

[76] Renthal W, Kumar A, Xiao G, Wilkinson M, Covington HE (2009). Genome-wide analysis of chromatin regulation by cocaine reveals a role for sirtuins.. Neuron.

[77] Renthal W, Nestler EJ (2009). Histone acetylation in drug addiction.. Semin Cell Dev Biol.

[78] Khan AN, Tomasi TB (2008). Histone deacetylase regulation of immune gene expression in tumor cells.. Immunol Res.

[79] Shahbazian MD, Grunstein M (2007). Functions of site-specific histone acetylation and deacetylation.. Annu Rev Biochem.

[80] Huang Y, Myers SJ, Dingledine R (1999). Transcriptional repression by REST: recruitment of Sin3A and histone deacetylase to neuronal genes.. Nat Neurosci.

[81] Naruse Y, Aoki T, Kojima T, Mori N (1999). Neural restrictive silencer factor recruits mSin3 and histone deacetylase complex to repress neuron-specific target genes.. Proc Natl Acad Sci U S A.

[82] De Cesare D, Sassone-Corsi P (2000). Transcriptional regulation by cyclic AMP-responsive factors.. Prog Nucleic Acid Res Mol Biol.

[83] Sng JC, Taniura H, Yoneda Y (2005). Inhibition of histone deacetylation by trichostatin A intensifies the transcriptions of neuronal c-fos and c-jun genes after kainate stimulation.. Neurosci Lett.

[84] Fischle W, Wang Y, Allis CD (2003). Histone and chromatin cross-talk.. Curr Opin Cell Biol.

[85] Winter S, Fischle W (2010). Epigenetic markers and their cross-talk.. Essays Biochem.

[86] Murr R (2010). Interplay between different epigenetic modifications and mechanisms.. Adv Genet.

[87] Wang Z, Zang C, Cui K, Schones DE, Barski A (2009). Genome-wide mapping of HATs and HDACs reveals distinct functions in active and inactive genes.. Cell.

[88] Cadet JL, Brannock C, Krasnova IN, Ladenheim B, McCoy MT (2010). Methamphetamine-induced dopamine-independent alterations in striatal gene expression in the 6-hydroxydopamine hemiparkinsonian rats.. PLoS One.

[89] Cadet JL, Brannock C, Ladenheim B, McCoy MT, Beauvais G (2011). Methamphetamine preconditioning causes differential changes in striatal transcriptional responses to large doses of the drug.. Dose Response.

[90] McCoy MT, Jayanthi S, Wulu JA, Beauvais G, Ladenheim B (2011). Chronic methamphetamine exposure suppresses the striatal expression of members of multiple families of immediate early genes (IEGs) in the rat: normalization by an acute methamphetamine injection.. Psychopharmacology (Berl).

