# Supplementary figures and images for: Methamphetamine Causes Differential Alterations in Gene Expression and Patterns of Histone Acetylation/Hypoacetylation in the Rat Nucleus Accumbens
Source: PLoS One. 2012 Mar 28;7(3):e34236. doi: 10.1371/journal.pone.0034236 (PMC3314616; doi:10.1371/journal.pone.0034236)

## Slide 1
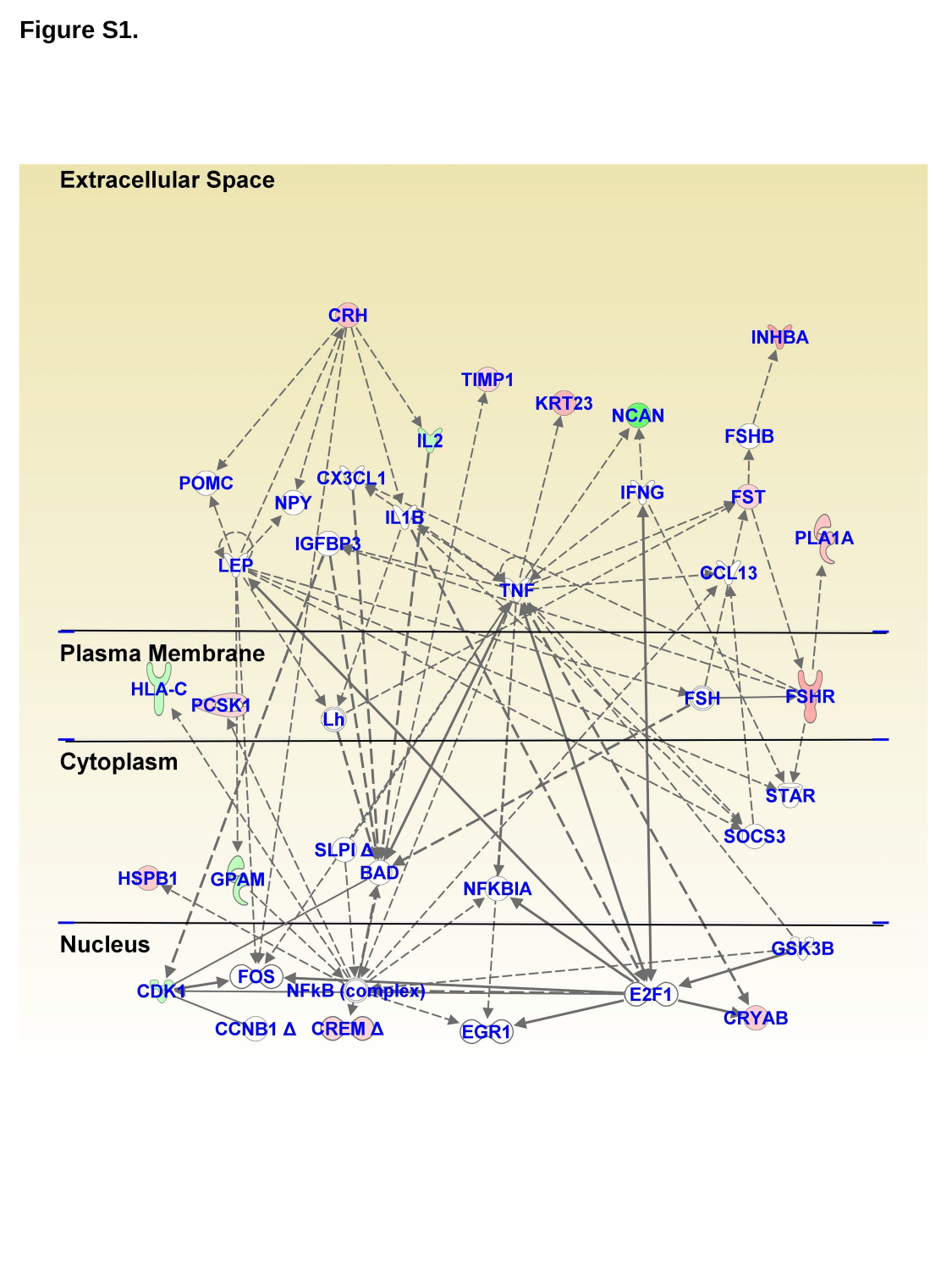

Figure S1.

Supplement: Figure S1 — A network of genes whose expression was affected by METH at 8-hr after the injection of the drug. Networks of related genes were identified using Ingenuity Pathway Analysis (IPA) software. The figure shows a network of affected genes that are involved in lipid metabolism, molecular transport, and cellular compromise and cell death. Color schemes are as described in figure 2. (PPT) [file pone.0034236.s001.ppt]

## Slide 1
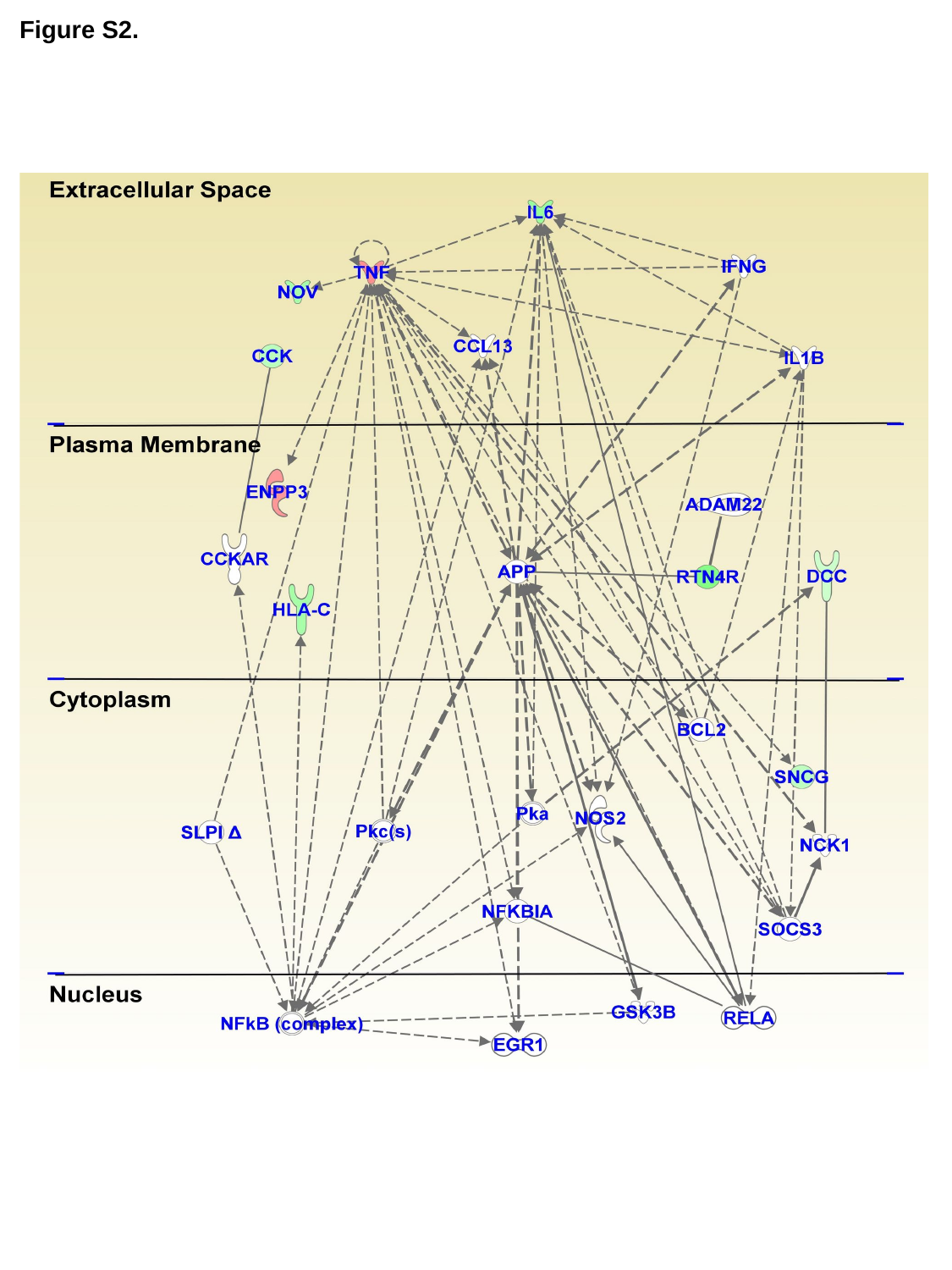

Figure S2.

Supplement: Figure S2 — A network of genes whose expression was affected by METH at 16-hr after the injection of the drug. Networks of related genes were identified using Ingenuity Pathway Analysis (IPA) software. The figure shows a network of genes that participate in cellular development, cellular growth and nervous system development. Relationships are shown as lines and arrows. Color schemes are as described in figure 2. (PPT) [file pone.0034236.s002.ppt]

## Slide 1
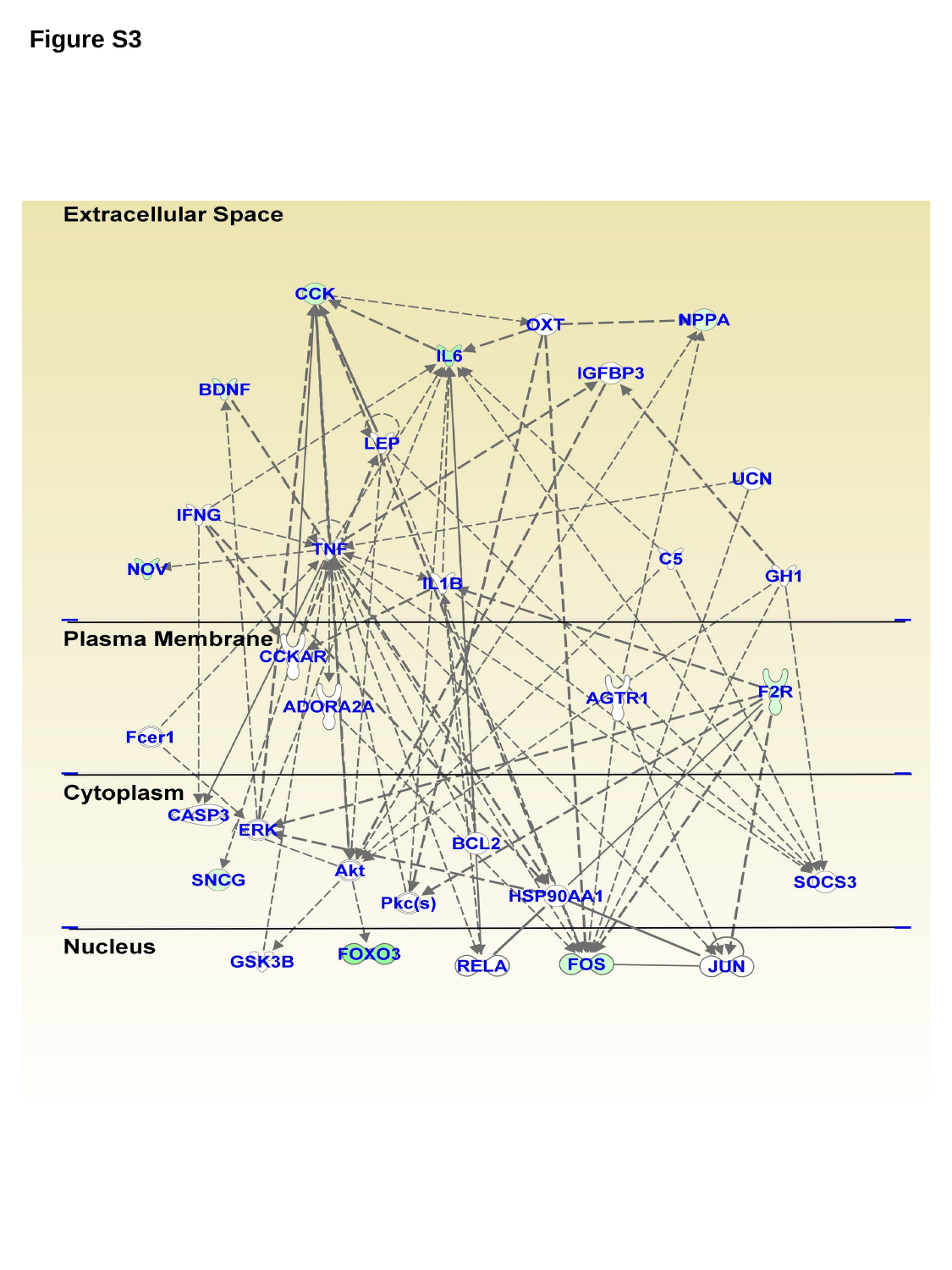

Figure S3

Supplement: Figure S3 — A network of genes whose expression was affected by METH at 24-hr after injection of the drug. Networks of related genes were identified using Ingenuity Pathway Analysis (IPA) software. The figure shows genes involved in the regulation of cell death, nervous system development, and cell proliferation. Relationships are shown as lines and arrows. Color schemes are as described in figure 2. (PPT) [file pone.0034236.s003.ppt]

## Slide 1
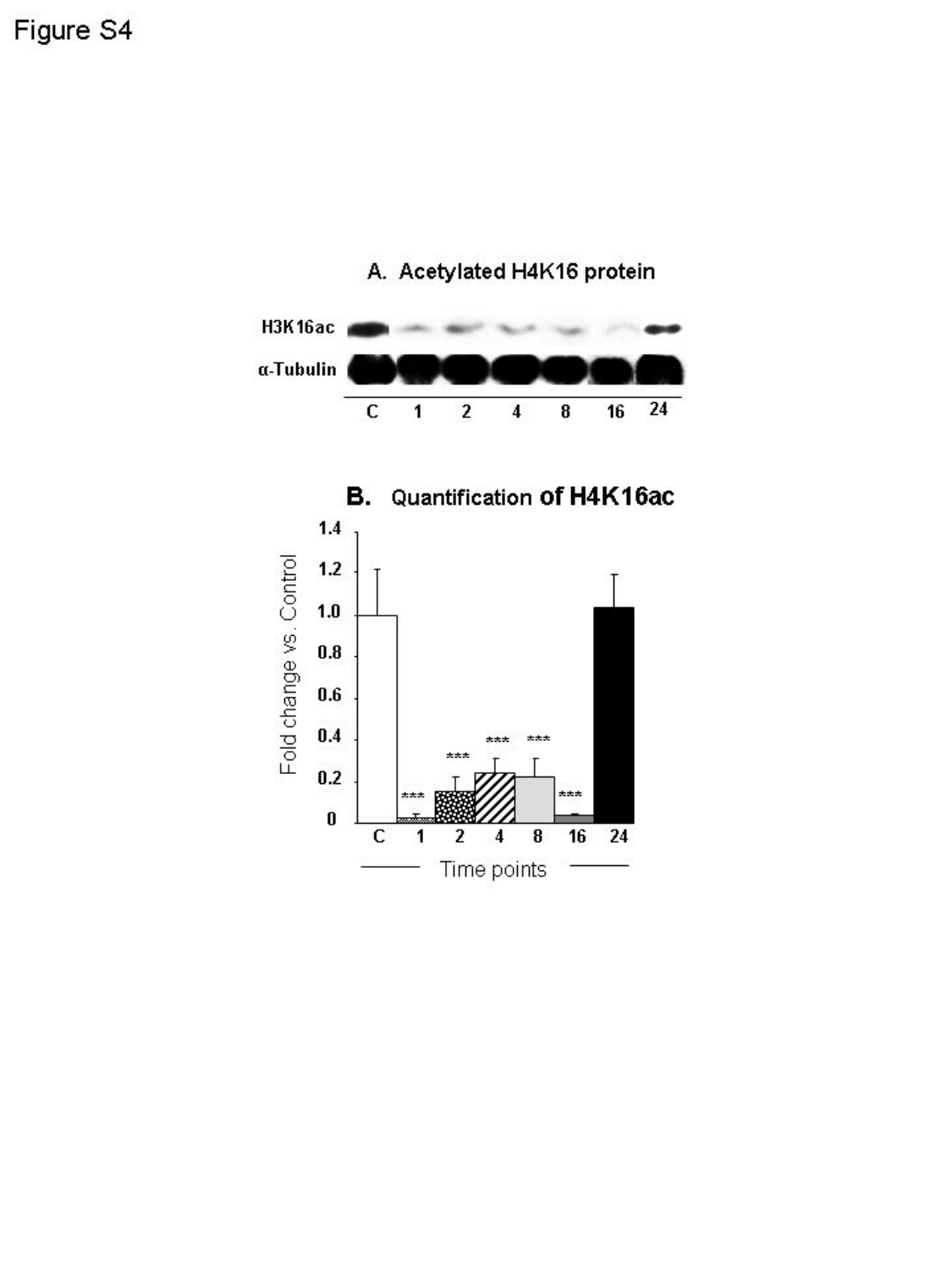

Supplement: Figure S4 — METH administration caused significant decreases in H4K16 acetylation in the NAC. The graph shows representative results from Western blot analyses using a specific antibody against (A) H4K16ac at various time points after the injection of the drug. Western blot analyses and statistical analyses were carried out as described in Fig. 4. The bar graph shows quantification of the effects of METH on H4K16ac. Key to statistics: * = p<0.05; ** = p<0.01; *** = p<0.001, in comparison to the control group. (PPT) [file pone.0034236.s004.ppt]
