# Supplementary material for: Methamphetamine Causes Differential Alterations in Gene Expression and Patterns of Histone Acetylation/Hypoacetylation in the Rat Nucleus Accumbens
Source: PLoS One. 2012 Mar 28;7(3):e34236. doi: 10.1371/journal.pone.0034236 (PMC3314616; doi:10.1371/journal.pone.0034236)
Supplement: Table S1 — Partial list of METH-upregulated genes measured at 1-hr after the drug injection. The list of genes was generated as described in the text. The genes are listed in descending order according to METH-induced fold changes in transcript levels. (DOC) [file pone.0034236.s005.doc]

**Table S1. Partial list of METH-upregulated genes measured at 1-hr after the drug injection**

| **Gene Symbol** | **Definition** | **1h** |
| --- | --- | --- |
| Npas4 | neuronal PAS domain protein 4 | 28.97 |
| c-fos | FBJ murine osteosarcoma viral oncogene homolog | 28.67 |
| Irs2 | insulin receptor substrate 2 | 18.65 |
| Creb3l1 | cAMP responsive element binding protein 3-like 1 | 15.83 |
| Slpi | secretory leukocyte peptidase inhibitor | 14.34 |
| Egr4 | early growth response 4 | 8.34 |
| Nr4a3 | nuclear receptor subfamily 4, group A, member 3 | 8.07 |
| Egr2 | early growth response 2 | 7.42 |
| Nr4a2 | nuclear receptor subfamily 4, group A, member 2 | 6.35 |
| Dusp1 | dual specificity phosphatase 1 | 6.18 |
| Junb | Jun-B oncogene | 5.83 |
| Crh | corticotropin releasing hormone | 5.72 |
| Cyr61 | cysteine rich protein 61 | 5.45 |
| Nfil3 | nuclear factor, interleukin 3 regulated | 4.46 |
| Cebpb | CCAAT/enhancer binding protein (C/EBP), beta | 4.16 |
| Gadd45g | growth arrest and DNA-damage-inducible 45 gamma | 3.97 |
| Il6ra | interleukin 6 receptor, alpha | 3.86 |
| Nptx2 | neuronal pentraxin II | 3.75 |
| Egr1 | early growth response 1 | 3.43 |
| Homer2 | homer homolog 2 (Drosophila) | 3.01 |
| Plekhf1 | pleckstrin homology domain containing, family F member 1 | 2.76 |
| Uap1 | UDP-N-acetylglucosamine pyrophosphorylase 1 | 2.74 |
| Kcnf1 | potassium voltage-gated channel, subfamily F, member 1 | 2.70 |
| Jun | Jun oncogene | 2.67 |
| Arf4l | ADP-ribosylation factor 4-like | 2.64 |
| Dusp6 | dual specificity phosphatase 6 | 2.58 |
| Per1 | period homolog 1 (Drosophila) | 2.56 |
| Stx1a | syntaxin 1A (brain) | 2.44 |
| Rasl11a | RAS-like family 11 member A | 2.41 |
| Nfkbia | nuclear factor of kappa light chain gene enhancer in B-cells inhibitor, alpha | 2.37 |
| Dnajb1 | DnaJ (Hsp40) homolog, subfamily B, member 1 | 2.26 |
| Bcl6 | B-cell leukemia/lymphoma 6 | 2.26 |
| Ngfg | nerve growth factor, gamma | 2.19 |
| Sgk | serum/glucocorticoid regulated kinase | 2.19 |
| Ddit4 | DNA-damage-inducible transcript 4 | 2.09 |
| Gadd45b | growth arrest and DNA-damage-inducible 45 beta | 1.83 |
| Fgf12 | fibroblast growth factor 12 | 1.76 |
| Atf3 | activating transcription factor 3 | 1.73 |

The list of genes was generated as described in the text. The genes are listed in descending order according to METH-induced fold changes in transcript levels.
