# Supplementary material for: Methamphetamine Causes Differential Alterations in Gene Expression and Patterns of Histone Acetylation/Hypoacetylation in the Rat Nucleus Accumbens
Source: PLoS One. 2012 Mar 28;7(3):e34236. doi: 10.1371/journal.pone.0034236 (PMC3314616; doi:10.1371/journal.pone.0034236)
Supplement: Table S2 — Partial list of METH-regulated genes measured at 8-hr after the drug injection. The genes are listed in descending order according to METH-induced fold changes in gene expression at the 8-hr. time point. The values for the 16- and 24-hr time points are listed for comparison. (DOC) [file pone.0034236.s006.doc]

**Table S2. Partial list of METH-regulated genes measured at 8-hr after the drug injection**

| **Gene** | **Definition** | **8h** | **16h** | **24h** |
| --- | --- | --- | --- | --- |
| Dusp14 | dual specificity phosphatase 14 | **9.66** | 1.02 | 3.29 |
| Dok3 | docking protein 3 | **5.24** | 1.99 | 1.29 |
| Smad6 | MAD homolog 6 (Drosophila) | **5.03** | 1.78 | -1.69 |
| Hcrtr1 | hypocretin (orexin) receptor 1 | **4.94** | 4.04 | 1.62 |
| Inhba | inhibin beta-A (Inhba), mRNA | **4.12** | 1.21 | 1.81 |
| Fshr | follicle stimulating hormone receptor | **3.91** | 1.16 | 2.90 |
| Pspn | persephin | **3.69** | 1.12 | 1.08 |
| Nfkbil2 | nuclear factor of kappa light polypeptide gene enhancer in B-cells inhibitor-like 2 | **3.66** | 2.11 | 1.80 |
| Ticam2 | toll-like receptor adaptor molecule 2 | **3.58** | 1.14 | -1.46 |
| Ptch1 | patched homolog 1 | **3.42** | -2.12 | -2.00 |
| Angpt2 | angiopoietin 2 | **3.34** | -1.49 | -1.23 |
| Tac4 | tachykinin 4 | **3.34** | -1.15 | 1.02 |
| Sp7 | Sp7 transcription factor | **3.32** | -1.53 | -1.04 |
| Pmaip1 | phorbol-12-myristate-13-acetate-induced protein 1 | **3.25** | -1.10 | 1.13 |
| Glipr1 | GLI pathogenesis-related 1 (glioma) | **3.16** | 1.14 | 1.60 |
| Crh | corticotropin releasing hormone | **2.90** | 1.92 | 1.27 |
| Nmu | Neuromedin U | **2.87** | 2.00 | 1.28 |
| Pla1a | phospholipase A1 member A | **2.75** | 1.35 | 1.13 |
| Rag1 | recombination activating gene 1 | **2.74** | 2.20 | 1.20 |
| Sv2c | synaptic vesicle glycoprotein 2c | **2.74** | 1.83 | 1.28 |
| Serpina9 | serine (or cysteine) peptidase inhibitor | **2.64** | 2.14 | 1.41 |
| Hspb1 | heat shock 27kDa protein 1 | **2.44** | 1.08 | -1.07 |
| Nts | neurotensin | **2.38** | 1.54 | -1.09 |
| Cryab | crystallin, alpha B | **2.35** | 1.13 | 1.05 |
| Ret | ret proto-oncogene | **2.35** | 1.54 | -1.34 |
| Tmem45b | transmembrane protein 186 | **2.35** | 1.44 | 1.15 |
| Cables1 | Cdk5 and Abl enzyme substrate 1 | **2.14** | -1.08 | 1.08 |
| Hist1h2ao | histone 1, H2ao | **2.00** | 1.08 | -1.13 |
| Pnoc | prepronociceptin | **2.00** | 1.52 | 1.18 |

The genes are listed in descending order according to METH-induced fold changes in gene expression at the 8-hr. time point. The values for the 16- and 24-hr time points are listed for comparison.
