# Supplementary material for: Methamphetamine Causes Differential Alterations in Gene Expression and Patterns of Histone Acetylation/Hypoacetylation in the Rat Nucleus Accumbens
Source: PLoS One. 2012 Mar 28;7(3):e34236. doi: 10.1371/journal.pone.0034236 (PMC3314616; doi:10.1371/journal.pone.0034236)
Supplement: Table S3 — Partial list of METH-regulated genes measured at 16-hr after the drug injection. The genes are listed in descending order according to METH-induced fold changes in gene expression at the 16-hr. time point. The values for the 8- and 24-hr time points are listed for comparison. (DOC) [file pone.0034236.s007.doc]

**Table S3. Partial list of METH-regulated genes measured at 16-hr after the drug injection**

| **Gene** |  | **8h** | **16h** | **24h** |
| --- | --- | --- | --- | --- |
| Sart1 | squamous cell carcinoma antigen recognized by T-cells 1 | 2.04 | **4.54** | 5.54 |
| Ttn | titin | -1.00 | **4.14** | 1.24 |
| Kctd11 | potassium channel tetramerisation domain containing 11 | 2.30 | **4.10** | 2.21 |
| Ppara | peroxisome proliferator activated receptor alpha | 1.38 | **3.90** | 1.18 |
| Gfpt1 | glutamine fructose-6-phosphate transaminase 1 | 3.08 | **3.85** | 2.22 |
| Tll1 | tolloid-like 1 | 1.15 | **3.63** | -1.04 |
| Hs6st1 | heparan sulfate 6-O-sulfotransferase 1 | 1.94 | **3.40** | 1.85 |
| Tnf | tumor necrosis factor superfamily, member 2 | 1.90 | **3.24** | 1.31 |
| Slc25a34 | solute carrier family 25, member 34 | 3.38 | **3.20** | 2.48 |
| Enpp3 | ectonucleotide pyrophosphatase/phosphodiesterase 3 | -1.00 | **3.02** | -1.00 |
| Nkx2-3 | NK2 transcription factor related, locus 3 (Drosophila) | 1.65 | **2.82** | 1.85 |
| Runx3 | runt-related transcription factor 3 | 1.51 | **2.81** | 1.69 |
| Kap | kidney androgen regulated protein | 1.78 | **2.58** | 1.33 |
| Stk40 | serine/threonine kinase 40 | 2.85 | **2.55** | 2.09 |
| Hist1h1t | histone cluster 1, H1t | 2.10 | **2.44** | 1.44 |
| Aim1 | absent in melanoma 1 | -1.18 | **2.32** | -1.49 |
| Unc45b | unc-45 homolog B (C elegans) | 1.10 | **2.20** | -1.00 |
| Amhr2 | anti-Mullerian hormone type 2 receptor | 1.57 | **2.16** | 1.25 |
| Ung | uracil-DNA glycosylase | 1.10 | **2.15** | 1.73 |
| Rilp | Rab interacting lysosomal protein | 1.04 | **2.05** | -1.05 |
| Nmu | neuromedin U | 2.87 | **2.00** | 1.28 |
| Allc | allantoicase | 1.28 | **1.96** | 1.71 |
| Thrsp | thyroid hormone responsive protein | -1.69 | **-1.95** | -1.29 |
| Prss12 | protease, serine, 12 neurotrypsin (motopsin) | -1.76 | **-1.98** | -1.66 |
| Mmrn2 | multimerin 2 | 1.02 | **-1.98** | -1.79 |
| Dcc | deleted in colorectal carcinoma | -2.84 | **-2.06** | -2.07 |
| Cck | cholecystokinin | -2.14 | **-2.67** | -2.79 |
| Cpa3 | carboxypeptidase A3 | -1.76 | **-2.17** | -3.39 |
| Sncg | synuclein, gamma | -1.19 | **-2.58** | -1.94 |
| Trp53inp2 | TRAF3 interacting protein 3 | -1.23 | **-2.73** | -1.32 |
| Cdh3 | cadherin 3, type 1, P-cadherin (placental) | -1.68 | **-2.75** | -3.33 |
| Vwa2 | von Willebrand factor A domain containing 2 | -1.93 | **-2.86** | -1.53 |
| Samsn1 | SAM domain, SH3 domain and nuclear localization signals, 1 | 1.75 | **-3.35** | 2.01 |
| Chaf1b | chromatin assembly factor 1, subunit B (p60) | -1.98 | **-3.87** | -2.86 |
| Chrna4 | cholinergic receptor, nicotinic | 1.06 | **-4.32** | -1.76 |
| Gpr143 | G protein-coupled receptor 143 | -1.99 | **-5.35** | -1.90 |
| Neurod1 | neurogenic differentiation 1 | -2.64 | **-5.48** | -3.71 |

The genes are listed in descending order according to METH-induced fold changes in gene expression at the 16-hr. time point. The values for the 8- and 24-hr time points are listed for comparison.
