# Supplementary material for: Methamphetamine Causes Differential Alterations in Gene Expression and Patterns of Histone Acetylation/Hypoacetylation in the Rat Nucleus Accumbens
Source: PLoS One. 2012 Mar 28;7(3):e34236. doi: 10.1371/journal.pone.0034236 (PMC3314616; doi:10.1371/journal.pone.0034236)
Supplement: Table S4 — Partial list of METH- regulated genes measured at 24-hr after the drug injection. The genes are listed in descending order according to METH-induced fold changes in gene expression at the 24-hr. time point. The values for the 8- and 16-hr time points are listed for comparison. (DOC) [file pone.0034236.s008.doc]

**Table S4. Partial list of METH- regulated genes measured at 24-hr after the drug injection**

| **Gene** | **Definition** | **8h** | **16h** | **24h** |
| --- | --- | --- | --- | --- |
| Nalp10 | NACHT, leucine rich repeat and PYD containing 10 | 1.06 | -1.28 | **6.53** |
| Ntng2 | netrin G2 | 1.39 | 1.56 | **5.16** |
| Kcnk18 | potassium channel, subfamily K, member 18 | 1.98 | 1.14 | **4.48** |
| Rad51ap1 | RAD51 associated protein 1 | 2.76 | 1.13 | **4.37** |
| V1rm1 | vomeronasal V1r-type receptor V1rm1 | 1.50 | 1.07 | **3.87** |
| Ca5a | carbonic anhydrase 5a | 1.95 | 1.66 | **3.38** |
| Foxd2 | forkhead box D2 | 1.16 | 1.25 | **3.27** |
| Pde6b | phosphodiesterase 6B, cGMP, rod receptor, beta polypeptide | 1.36 | 1.29 | **3.22** |
| Il12rb1 | interleukin 12 receptor, beta 1 | -1.00 | 1.04 | **3.05** |
| Ddx26 | DEAD/H (Asp-Glu-Ala-Asp/His) box polypeptide 26 | 1.81 | 1.24 | **2.77** |
| RT1-CE4 | RT1 class I, CE4 | 1.55 | 1.33 | **2.34** |
| Armc3 | armadillo repeat containing 3 | 1.19 | 1.87 | **2.25** |
| Sgcg | sarcoglycan, gamma (dystrophin-associated glycoprotein) | -1.06 | 1.28 | **2.06** |
| Acadsb | acyl-Coenzyme A dehydrogenase, short/branched chain | -1.17 | -1.85 | **-1.96** |
| Slc10a7 | solute carrier family 10 (sodium/bile acid cotransporter family) | -1.52 | -1.08 | **-1.96** |
| Lin7c | lin-7 homolog C (C elegans) | -1.15 | -1.07 | **-1.96** |
| F2r | coagulation factor II (thrombin) receptor | -1.19 | 1.02 | **-2.01** |
| Vip | vasoactive intestinal polypeptide | -1.42 | -1.85 | **-2.01** |
| Matp | membrane associated transporter protein | -1.37 | -1.15 | **-2.24** |
| Tspyl5 | TSPY-like 5 | -1.50 | 1.09 | **-2.33** |
| Fos | FBJ murine osteosarcoma viral oncogene homolog | 1.20 | 1.27 | **-2.46** |
| Tmigd | transmembrane and immunoglobulin domain containing | -1.39 | -1.22 | **-2.73** |
| Csnk1g1 | casein kinase 1, gamma 1 | -1.90 | -2.27 | **-2.90** |
| Kcnh7 | potassium voltage-gated channel, subfamily H | -1.17 | 1.03 | **-2.91** |
| Ela2a | elastase 2A | -1.78 | -2.10 | **-3.20** |
| RT1-M1-2 | RT1 class I, M1, gene 2 | -2.29 | -1.73 | **-3.39** |
| Umod | uromodulin | -1.25 | -2.54 | **-3.53** |
| Slc16a14 | solute carrier family 16 (monocarboxylic acid transporters) | -1.35 | -1.81 | **-3.53** |
| Glb1l3 | galactosidase, beta 1-like 3 | -1.58 | -2.56 | **-3.56** |
| Pcdh1 | protocadherin 1 (cadherin-like 1) | -1.10 | 1.02 | **-3.78** |
| Twsg1 | twisted gastrulation homolog 1 (Drosophila) | -2.25 | -2.96 | **-3.86** |
| Plac1 | placenta-specific 1 | -3.19 | 1.16 | **-3.88** |
| Stk36 | serine/threonine kinase 36 (fused homolog, Drosophila) | -1.35 | -3.15 | **-3.96** |
| Foxo3 | forkhead box O3 | -1.87 | -1.12 | **-5.19** |
| Sla | src-like adaptor | -3.13 | -3.01 | **-5.61** |
| Slc23a2 | solute carrier family 23 (nucleobase transporters), member 2 | -2.52 | -1.78 | **-6.10** |
| Npas4 | neuronal PAS domain protein 4 | 1.20 | 1.07 | **-6.57** |
| Plekha6 | pleckstrin homology domain containing, family A member 6 | -2.20 | -3.31 | **-9.59** |

The genes are listed in descending order according to METH-induced fold changes in gene expression at the 24-hr. time point. The values for the 8- and 16-hr time points are listed for comparison.
